# Predictors of Outcomes of Parent Training Targeting Disruptive Behavior in Children Aged 4 Years at 6-Month Follow-Up: Results From a Large Prospective Cohort Implementation Study

**DOI:** 10.2196/79592

**Published:** 2026-04-28

**Authors:** Minja Westerlund, Susanna Hinkka-Yli-Salomäki, Terja Ristkari, Malin Kinnunen, Amit Baumel, Andre Sourander

**Affiliations:** 1 Research Centre for Child Psychiatry, Faculty of Medicine, University of Turku, Lemminkäisenkatu 3a, Teutori, 3rd floor, Turku, 20520, Finland, 358 50 365 3447; 2 INVEST Research Flagship Centre, University of Turku, Turku, Finland; 3 Department of Community Mental Health, University of Haifa, Haifa, Israel; 4 Department of Child Psychiatry, Turku University Hospital, Turku, Finland

**Keywords:** parent training, implementation, digital interventions, psychosocial, children

## Abstract

**Background:**

Parent training interventions effectively reduce disruptive behavior in children. However, research on how participant characteristics and program factors influence the outcomes in real-world settings remains scarce.

**Objective:**

This study aimed to identify factors predicting outcomes of the internet-based, telephone-assisted Strongest Families Parent training program.

**Methods:**

A prospective cohort implementation study conducted within population-based screening embedded in routine health checkups targeting all children aged 4 years in Finland, to identify children with high levels of conduct problems and functional impairment. From a study population of 49,504, altogether 3911 participants completed baseline measures, 707 participants did not do so at 6 months follow-up, resulting in a sample of 3204 (1158/3186, 36.3% girl, 2028/3186, 63.7% boys). Reported duration of difficulties was 6 months in 29.57% (934/3159) of participants, 6‐12 months in 27% (853/3159) of participants, and >12 months in 43.43% (1372/3159) of participants. Most children lived with 2 biological parents (2721/3194, 85.19%). A total of 35.24% (1121/3181) of mothers and 26.18% (797/3044) of fathers had a university degree. Data was collected via parent report. Multinomial logistic regression analyses were conducted to identify which child-, family-, and program-related factors predicted changes in the Child Behavior Checklist 1.5-5 (CBCL) externalizing subscale from baseline to 6-month follow-up. The standardized change in CBCL externalizing score was created by subtracting the mean at baseline from the individual 6-month measurement, divided by the SD at baseline. The standardized change was categorized to ±0.5 SD (no change); +0.5 to +1.5 SD (moderate improvement), >+1.5 SD (large improvement), and more than −0.5 SD (deterioration). A *P* value of <.05 was considered significant.

**Results:**

In 77% (2468/3204) of participants, symptoms improved at 6-month follow-up. Multinomial logistic regression analyses with α-level of <0.05 showed that >12 months duration of initial problems, callous-unemotional traits, and CBCL internalizing symptoms were linked to lower likelihood of large improvement (odds ratio [OR] 0.43, 95% CI 0.33‐0.56; *P*<.001; OR 0.64, 95% CI 0.57‐0.73; *P*<.001; OR 0.54, 95% CI 0.47‐0.63; *P*<.001, respectively). Definite and severe problems at baseline were linked to deterioration (OR 2.29, 95% CI 1.62‐3.24; *P*<.001; OR 4.38, 95% CI 2.80‐6.85; *P*<.001, respectively). Parental stress was linked to a lower likelihood of large improvement (OR 0.78, 95% CI 0.67‐0.91; *P*=.002), and anxiety to a higher likelihood of deterioration (OR 1.20, 95% CI 1.04‐1.39; *P*=.02).

**Conclusions:**

Children with longer-term and more severe behavioral symptoms may require tailored intervention. Support for parents with stress may be recommended. Much of the current literature on parent training is based on randomized controlled trials, while the literature on the implementation of parenting programs and studies examining change is limited. Our study informs about predictors of treatment outcomes when interventions are implemented. These results are important clinically as they allow personalization of interventions.

## Introduction

Randomized controlled trials (RCTs) have offered robust empirical support for the efficacy of parent training in reducing disruptive behavior in children [1,2]. However, as widely acknowledged by clinicians, family responses to parent training interventions, even when they are empirically supported, can vary considerably [3]. There have been calls for studies evaluating psychosocial interventions outside of structured research settings, using larger and more heterogeneous samples than are typically used in RCTs [4]. An ideal opportunity for conducting such studies would be when interventions are implemented in practice and used by substantial portions of the target population, but such large-scale implementation studies are not routinely carried out. Consequently, the literature on the implementation of parenting programs in community settings is scarce [5-7]. Much of the current evidence is based on RCTs with limited sample sizes and often clinical samples, limiting a comprehensive examination of the predictors of treatment outcomes in real-life settings. Implementation research can provide a basis for more personalized care by identifying [8] which interventions are most effective for which individuals [9].

Multiple factors may influence the efficacy of parent training programs [10]. However, much of the existing evidence is derived from RCTs with rather small sample sizes, and only a limited number of studies have been conducted in implementation settings [11,12]. Further, few have used population-based screening methods [13,14] or have assessed digital parent training interventions [15-18]. Among the various predicting characteristics of children, younger age has been associated with the likelihood of benefitting from parent training programs [19], including when such programs are delivered in digital format [20]. Higher initial severity of behavioral problems has been associated with greater reductions in problem behaviors following a digitally delivered parent training program [16]. Relatedly, another study [21] showed that callous-unemotional (callous-unemotional) traits among children (ie, individual characteristics marked by a lack of empathy, remorse, and guilt) reduced the functional gains of a parent-training program, especially when treatment was delivered digitally. Nevertheless, callous-unemotional traits were found to attenuate the program’s effects on problem severity and treatment response status. Regarding parental characteristics, a meta-analysis [22] found that low socioeconomic status or lower education level did not seem to diminish the effectiveness of parent training interventions, although it was suggested that the gained effects were more difficult to maintain over longer follow-up periods [16]. Findings related to parental psychopathology, and depression in particular, have been mixed, with some studies pointing to greater improvement in child disruptive behavior and others pointing to the opposite [10,23,24]. There is also some qualitative evidence indicating that family stressors such as illness or bereavement may be linked to a deterioration of parenting skills that are taught as part of a parent group training program for child conduct problems [25]. Finally, program-related factors associated with intervention success include program completion [16] as well as formats involving direct user engagement rather than formats that do not involve direct user engagement when assessing digital interventions [20].

Based on the demonstrated efficacy of the internet-based and telephone-assisted Finnish Strongest Families Parent (FSFP) training program in reducing children’s disruptive behavior both in RCT and population implementation settings [26,27], the present study aimed to identify factors that predict poor and favorable program outcomes when applied in a real-world setting. To address key gaps in the literature, we conducted this investigation within an implementation context, using population-based screening and a large sample size to assess factors potentially influencing the efficacy and adequacy of the FSFP. Specifically, we aimed to evaluate the outcomes at 6-month follow-up in families who had completed the FSFP, to assess the distribution of the outcomes, and to identify factors associated with these different outcomes.

## Methods

### Study Design

The participants (n=3204) were derived from an implementation study spanning over 100 child health clinics in 15 administrative regions in Finland. All families who participated in the FSFP implementation study and who provided data at 6 months follow-up were included in this study. The families were originally recruited to the FSFP program between February 8, 2015, and October 9, 2022. The target group comprised children aged 4 years attending routine medical check-ups at primary care child health clinics. Child health clinic services are free primary health care services provided to families with children under school age in Finland. At least 15 child health check-ups are offered with intervals varying from weeks in infancy to months later and about a year before school age. Before the health check-up, the families received the screening survey by post and were requested to complete it and bring it along to the health check-up. Participants who had not completed the survey were offered the opportunity to do so at the consultation. This screening survey was collected in paper format and included demographic information and the Strengths and Difficulties Questionnaire (SDQ). We requested the participants to complete the baseline (Child Behavior Checklist 1.5-5 [CBCL], Inventory of Callous-Unemotional Traits [ICU], Parenting Scale [PS], 21-Item Depression, Anxiety and Stress Scale [DASS-21]; see Measures) and follow-up CBCL measures online.

**Figure 1.**
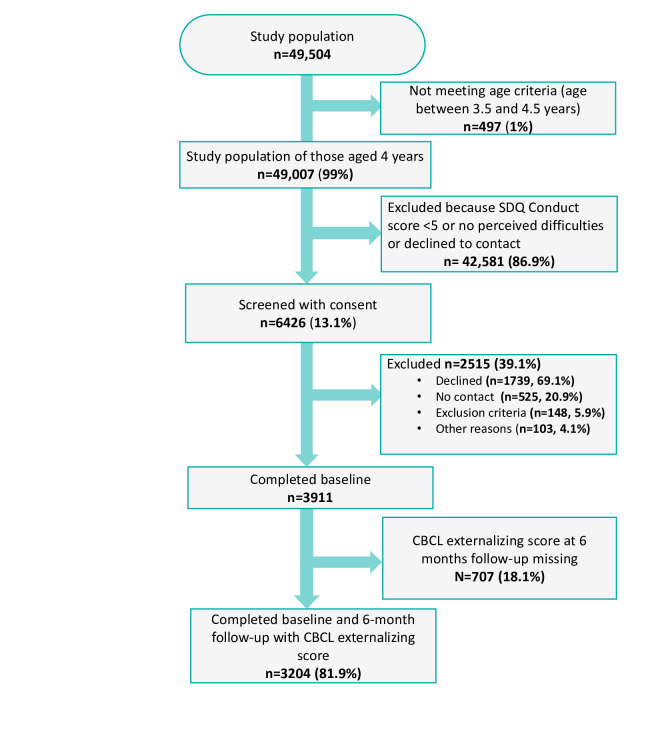
Flow chart of the screening procedures. CBCL: Child Behavior Checklist 1.5-5; SDQ: Strengths and Difficulties Questionnaire.

From a study population of 49,504, a total of 6426 (13.1%) families met the screening criteria and consented (Figure 1). The screening criteria were a score of ≥5 on the conduct scale of SDQ [28], and at least minor difficulties in mood, concentration, behavior, or getting along with other people over the past 6 months, according to parental report, and a willingness to be contacted by this study’s team. The cutoff score of ≥5 was based on the SDQ conduct score 80th percentile cutoff point in a population-based sample of 931 Finnish children aged 4 years [26,29].

A total of 3911 participants eligible for the FSFP completed the baseline measure. Further, 707 (18.1%) participants did not complete the CBCL externalizing measure at 6 months follow-up, which resulted in a total of 3204 (81.9%) participants meeting the inclusion criteria for this study assessing predictors of FSFP outcomes at 6-month follow-up. The participants had to meet the abovementioned screening criteria; have access to a telephone and internet at home, have fluency in Finnish or Swedish by at least 1 parent, residence in one of the administrative regions participating in the program, and a completed 6-month CBCL externalizing score. Exclusion criteria included autism, Down syndrome, fetal alcohol syndrome, an intellectual disability, or severe mental disorders.

### Ethical Considerations

This study received ethical approval from the Ethics Committee of the University of Turku (drno. 44/2014 and 25/2018). Study permission was given by local administrations from participating regions in Finland. Written informed consent for this study was obtained from parents participating in this study, and they were free to decline participation in this study at any time. This study’s data were deidentified by replacing personal information with a study subject code. The ID code key is kept separate from other research data. Participants did not receive any kind of compensation, monetary or otherwise. No identifying information or images of individual participants are included in this paper or its supplementary materials.

### Intervention

The FSFP parent training program aims to strengthen skills to improve parent-child relationships. Important elements of the program include supporting parents in recognizing positive behaviors in their child and reinforcing those behaviors by responding to them with positive methods. The 11-week program consists of web-based materials (eg, psychoeducational material, videos, and exercises) and weekly telephone coaching by trained family coaches (licensed health care or social work professionals). Telephone coaching allows for a goal-oriented and personalized approach to solving the challenges that the family is experiencing. The family coach guides the parent in practicing skills that strengthen their parenting, such as positive interaction and rewarding, sharing attention, preempting transitions, planning everyday situations, collaborating with daycare staff, and managing restraint of the child [26,27]. Each week consists of a specific theme, which the parents complete before progressing to the following theme. The last sessions focus on solidifying the newly learned positive parenting skills. The web-based material consists of an introduction, the session content, video exercises, troubleshooting, review, and practical application of the new skills. Each telephone coaching session is scheduled at the conclusion of the preceding weekly call and is allotted a duration of 1 hour. Missed appointments are followed up by the family coach.

### Implementation

The FSFP was implemented in the various regions of the Finnish health care service system starting in 2015. The implementation plan included staff recruitment, training, supervision, performance evaluation, and administrative support. Given that the fidelity of an intervention delivery may be compromised when transferring it from a controlled research context to a community setting [30], the FSFP program uses a centralized treatment provider unit, the Research Center for Child Psychiatry at the University of Turku. The coaches are trained on the digital program protocol, and about 10% of calls are randomly audited for quality. Additional training is provided when needed.

FSFP has become part of routine child health clinic services, and families meeting the inclusion criteria are eligible to participate. Child health care nurses are kept informed about client progress, including screening summaries, consent updates, and treatment outcomes. Stakeholders in child and family services are engaged, sign annual research agreements, and receive monthly progress reports. Regular training and updates support the integration into primary care, and media campaigns have raised public awareness. Centralized delivery and development of the digital platform are key administrative strategies, ensuring high implementation fidelity—adherence to the strategy and adaptation to local practices.

### Measures

The demographic data included child’s sex (boy or girl), family structure (2 biological parents, 1 biological parent, or 2 nonbiological parents), parental age (<26 y/26–40 y/>40 y), and parental education (basic education, secondary education, or upper degree in applied sciences or university).

The CBCL [31] is a parent-reported measure of children’s emotional, behavioral, and social problems. It consists of 7 subscales: emotionally reactive, anxious or depressed, somatic complaints, withdrawn, sleep problems, attention problems, and aggressive behavior. The first 4 syndromes form an internalizing score, and the latter 2 an externalizing score. The externalizing score contains 24 items and was used to measure disruptive behavior in this study (ie, the primary outcome measure). Higher scores indicate a higher degree of problems. In the current sample, the internal consistencies were good for both the internalizing and externalizing subscales (0.86 and 0.87, respectively). For the analyses, we used the CBCL baseline and 6-month measures.

The ICU [32] is a 24-item scale used to measure callous-unemotional traits in children, where higher scores indicate a greater degree of callous-unemotional traits. The instrument has been shown to have good reliability and adequate validity for children aged 3-4 years, with a good internal consistency for the total score (0.93) [33]. In our sample, the Cronbach α for the ICU total score was 0.83. We used the ICU baseline measure in the analyses.

The PS was used to assess parenting skills [34]. This 30-item questionnaire measures 3 factors of dysfunctional parental discipline practices: laxness, overreactivity, and verbosity. Higher scores indicate a greater degree of dysfunctional parental discipline practices. The internal consistency for the total score was 0.77 in this study. We used the PS baseline measure in the analyses.

The Depression, Anxiety, and Stress Scale [35] was used to measure parents’ stress, anxiety, and depression symptoms. Higher scores indicate more symptoms. The short version of the instrument consists of 21 items (DASS-21) and has previously shown good internal consistency with Cronbach α of 0.91 (depression items), 0.80 (anxiety items), and 0.84 (stress items) in a large nonclinical sample of US adults [36]. The Cronbach α in our sample was 0.87, 0.75, and 0.84 for the depression, anxiety, and stress scales, respectively. We used the DASS-21 baseline measure in the analyses.

As for program measures, completion of at least 7 of the 11 program weeks was considered program completion, as the first 7 weeks of the intervention introduce the core elements of the intervention, while the final 4 weeks focus on reinforcing the acquired skills.

The program completion periods were defined as 2015‐2017, March 10, 2018, 2020 (ie, before the start of the COVID-19 pandemic), March 11, 2020, to December 31, 2020 (the acute COVID period, the World Health Organization declared the COVID-19 outbreak a global pandemic and many restrictions were in place), 2021 (the Finnish government stated that COVID-19 no longer constituted a state of emergency in Finland; vaccinations started on December 27, 2020), and 2022‐2023 (the end of restrictions and the post-COVID period).

### Analytic Strategy

The categorical family and child characteristics (as defined in the Measures paragraph) are presented as numbers and percentages, while the continuous program, family, and child characteristics are reported as means and SDs. The distributions of the CBCL externalizing scores at baseline (before starting the program) and at 6-month follow-up are presented using histograms. The outcome variable was derived from the CBCL externalizing score using the following formula:


$$Standardized\;\;change_{i}=\frac{Mean_{baseline}-measurement\;at\;6months}{SD_{baseline}},i=l,...,3204$$

In this formula, standardized change refers to the change in the CBCL externalizing score; mean (baseline) and SD (baseline) refer to the mean and SD of the CBCL externalizing score at baseline; and measurement at 6 months refers to each participant’s individual CBCL externalizing score at 6-month follow-up. Our derivation is a variant of a standard z-transformation, where our interest is to standardize each individual’s 6-month change, that is, how many SDs each individual deviates from the mean baseline, instead of calculating how many SDs each individual deviates from the mean at the same time point. Although the children participating in the FSFP had all elevated levels of disruptive behavior at baseline, variation in externalizing behavior levels was still high (quartile range 17‐27). Hence, children with more pronounced symptoms have more room for improvement, whereas for children with mild symptoms, only a small reduction of symptoms is possible, indicating a floor effect [37]. Therefore, we standardized the change in CBCL externalizing score by subtracting the mean at baseline from the 6-month measurement divided by the SD at baseline to compare individual changes more effectively. The standardized change was categorized so that a ±0.5 SD change indicated no change; +0.5 to 1.5 SD indicated a moderate improvement, and a change >1.5 SD indicated a large improvement. A negative change of more than −0.5 SD indicated a deterioration. We based our categorization on changes of 0.5 SD, which is an empirically derived criterion based on psychological theory [38,39] and established practice [40].

The associations of individual predictors of the outcome variable were analyzed with multinomial logistic regression models. Significant predictors were selected for the adjusted models based on a statistical significance level of 0.10. The point estimates are presented as odds ratios with 95% CIs and *P* values. A *P* value less than .05 was considered significant in the final model. The baseline scores of the psychometric scales were standardized before the analyses. All statistical analyses were performed with SAS (9.4 software; SAS Institute). In terms of analyses, the relationship of each predictor with the FSFP outcome was first examined; separate multinomial models of child characteristics and of parent or family characteristics were then performed to identify variables that remained associated within each domain. Finally, a combined multinomial analysis including child, parent, or family and program characteristics determined the independent predictors of the CBCL externalizing change categories. Multiple imputations were not performed as values were not missing at random.

## Results

**Figure 2.**
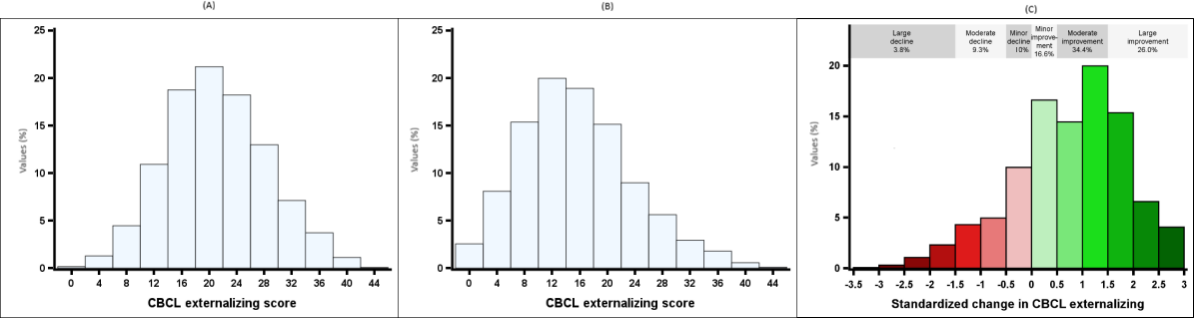
Distributions of CBCL externalizing (**A**) before starting the program (baseline), (**B**) at six months after starting the program, and (**C**) after standardization, where participants’ CBCL externalizing measurement at six months after starting the program was reduced from the baseline mean of the CBCL externalizing divided by its SD. CBCL: Child Behavior Checklist 1.5-5.

The mean CBCL at baseline was 21.6 (SD 7.3) and at 6 months 16.1 (SD 8.1). The mean change from baseline was 5.5 (SD 6.7), Cohen *d* 0.83. Altogether, 77% (2468/3204) of the participants showed a favorable change (ie, reduction of externalizing behavior) at 6-month follow-up. A total of 26% (833/3204) showed large (more than 1.5 SD) improvement, and 34.4% (1103/3204) showed moderate (0.5‐1.5 SD) improvement in the outcome variable (ie, change in CBCL externalizing). When the magnitude of change was smaller than 0.5 SD, the change was considered minor. A total of 16.6% (532/3204) experienced a minor improvement and 10% (319/3204) a minor decline. A total of 9.3% (297/3204) and 3.8% (120/3204) showed a moderate (−0.5 to −1.5 SD) or large (more than −1.5 SD) deterioration, respectively (Figure 2). Demographic and program information is presented in Table 1.

**Table 1.** Frequencies of child, parent, or family and program characteristics by the categorized standardized change in the CBCL^a^ externalizing score at 6 months after a parent training intervention targeting disruptive behavior in those aged 4 years in real-world settings, 2015‐2022. Missing observations per variable: sex (n=18), duration (n=45), adverse life events (n=239), family structure (n=10), maternal age (n=26), paternal age (n=117), maternal education (n=23), and paternal education (n=160).

Variable	All (n=3204)	Large improvement (n=833)	Moderate improvement (n=1103)	No change (n=851)	Moderate deterioration (n=297)	Large deterioration (n=120)
Child characteristics						
Sex, n (%)						
Female	1158 (36.35)	354 (11.1)	422 (13.25)	271 (8.51)	84 (2.64)	27 (0.85)
Male	2028 (63.65)	474 (14.9)	677 (21.25)	575 (18.05)	211 (6.62)	91 (2.86)
Difficulties, n (%)						
Minor	1407 (43.91)	527 (16.45)	537 (16.76)	287 (8.96)	49 (1.53)	7 (0.22)
Definite	1497 (46.72)	274 (8.55)	499 (15.57)	474 (14.79)	184 (5.74)	66 (2.06)
Severe	300 (9.36)	32 (1.00)	67 (2.09)	90 (2.81)	64 (2.00)	47 (1.47)
Duration of difficulties (months), n (%)						
6	934 (29.57)	347 (10.98)	346 (10.95)	178 (5.63)	48 (1.52)	15 (0.47)
6‐12	853 (27.0)	236 (7.47)	295 (9.34)	226 (7.15)	81 (2.56)	15 (0.47)
>12	1372 (43.43)	237 (7.50)	447 (14.15)	433 (13.71)	166 (5.25)	89 (2.82)
Adverse life events, n (%)						
None	2013 (67.89)	553 (18.65)	715 (24.11)	515 (17.37)	163 (5.50)	67 (2.26)
1	732 (24.69)	177 (5.97)	235 (7.93)	209 (7.05)	80 (2.70)	31 (1.05)
≥2	220 (7.42)	36 (1.21)	79 (2.66)	63 (2.12)	31 (1.05)	11 (0.37)
Psychometric scales*,* mean (SD)						
CBCL internalizing	−0.01 (0.98)	−0.46 (0.77)	−0.11 (0.89)	0.20 (0.96)	0.61 (1.14)	0.85 (1.18)
ICU^b^ score	−0.00 (0.99)	−0.40 (0.93)	−0.11 (0.90)	0.24 (0.94)	0.46 (0.94)	0.94 (1.07)
Family characteristics						
Family structure, n (%)						
2 biological parents	2721 (85.19)	729 (22.82)	952 (29.81)	714 (22.35)	239 (7.48)	87 (2.72)
One biological parent	362 (11.33)	74 (2.32)	114 (3.57)	102 (3.19)	50 (1.57)	22 (0.69)
Two nonbiological parents	111 (3.48)	28 (0.88)	35 (1.10)	30 (0.94)	8 (0.25)	10 (0.31)
Maternal age (years), n (%)						
<26	444 (13.97)	113 (3.56)	150 (4.72)	106 (3.34)	47 (1.48)	28 (0.88)
26‐40	2657 (83.61)	693 (21.81)	925 (29.11)	711 (22.37)	242 (7.61)	86 (2.71)
>40	77 (2.42)	21 (0.66)	19 (0.60)	26 (0.82)	6 (0.19)	5 (0.16)
Paternal age (years), n (%)						
<26	261 (8.45)	64 (2.07)	95 (3.08)	52 (1.68)	35 (1.13)	15 (0.49)
26‐40	2574 (83.38)	682 (22.09)	878 (28.44)	696 (22.55)	227 (7.35)	91 (2.95)
>40	252 (8.16)	58 (1.88)	94 (3.05)	69 (2.24)	21 (0.68)	10 (0.32)
Maternal education, n (%)						
Basic education	50 (1.57)	12 (0.38)	15 (0.47)	10 (0.31)	7 (0.22)	6 (0.19)
Secondary education	951 (29.90)	222 (6.98)	328 (10.31)	259 (8.14)	91 (2.86)	51 (1.60)
Upper degree in applied sciences	1059 (33.29)	287 (9.02)	374 (11.76)	271 (8.52)	92 (2.89)	35 (1.10)
University	1121 (35.24)	308 (9.68)	379 (11.91)	304 (9.56)	104 (3.27)	26 (0.82)
Paternal education, n (%)						
Basic education	144 (4.73)	41 (1.35)	46 (1.51)	36 (1.18)	16 (0.53)	5 (0.16)
Secondary education	1346 (44.22)	321 (10.55)	472 (15.51)	359 (11.79)	123 (4.04)	71 (2.33)
Upper degree in applied sciences	757 (24.87)	206 (6.77)	251 (8.25)	204 (6.70)	71 (2.33)	25 (0.82)
University	797 (26.18)	231 (7.59)	282 (9.26)	203 (6.67)	68 (2.23)	13 (0.43)
Psychometric scales, mean (SD)* *						
Parenting Scale	−0.02 (0.98)	−0.22 (0.99)	−0.02 (0.95)	0.13 (0.96)	0.21 (1.06)	−0.10 (0.95)
DASS^c^-depression	−0.05 (0.97)	−0.26 (0.82)	−0.09 (0.93)	0.04 (0.97)	0.36 (1.20)	0.23 (1.15)
DASS-anxiety	−0.03 (0.95)	−0.20 (0.80)	−0.08 (0.88)	0.01 (0.92)	0.37 (1.21)	0.35 (1.44)
DASS-stress	−0.03 (0.98)	−0.33 (0.93)	-0.06 (0.93)	0.08 (0.96)	0.45 (1.07)	0.30 (1.12)
Intervention characteristics						
Program completion, n (%)						
Completed <7 themes	70 (2.18)	19 (0.59)	29 (0.91)	16 (0.50)	1 (0.03)	5 (0.16)
Completed ≥7 themes	3134 (97.82)	814 (25.41)	1074 (33.52)	835 (26.06)	296 (9.24)	115 (3.59)
Period of completion, n (%)						
2015‐2017	482 (15.04)	135 (4.21)	183 (5.71)	114 (3.56)	33 (1.03)	17 (0.53)
2018 to March 10, 2020	975 (30.43)	240 (7.49)	334 (10.42)	260 (8.11)	108 (3.37)	33 (1.03)
March 11, 2020, to December 31, 2020	429 (13.39)	104 (3.25)	145 (4.53)	127 (3.96)	38 (1.19)	15 (0.47)
2021	531 (16.57)	131 (4.09)	172 (5.37)	154 (4.81)	49 (1.53)	25 (0.78)
2022‐2023	662 (20.66)	187 (5.84)	221 (6.90)	169 (5.27)	61 (1.90)	24 (0.75)

a CBCL: Child Behavior Checklist 1.5-5.

b ICU: Inventory of Callous-Unemotional Traits.

c DASS: 21-Item Depression, Anxiety and Stress Scale.

The relationships of each of the individual predictor variables of the outcome of the FSFP program are presented in Multimedia Appendix 1. The multinomial analysis (Table 2), including only child characteristics as predictors, showed that sex, internalizing symptoms, severity, and duration of problems, as well as callous-unemotional traits, were significantly associated with the different categories of the outcome. The multinomial analysis, including only parent or family characteristics, showed that maternal age >40 years, paternal age <26 years, mother’s education, parenting skills, parental anxiety, and stress were significantly associated with the outcome.

**Table 2.** Multivariable multinomial logistic regression analyses of the associations of child and family-level predictors, respectively, on the standardized change in CBCL^a^ externalizing as categorized into large improvement, moderate improvement, no change (reference group, n=851), and deterioration at 6 months after a parent training intervention targeting disruptive behavior in those aged 4 years in real-world settings, 2015‐2022. Missing observations per variable: sex (n=18), duration (n=45), adverse life events (n=239), family structure (n=10), maternal age (n=26), paternal age (n=117), maternal education (n=23), and paternal education (n=160).

Variable	Large improvement (n=833), OR^b^ (95% CI)	*P* value	Moderate improvement (n=1103), OR (95% CI)	*P* value	Deterioration (n=417), OR (95% CI)	*P* value
Child characteristics						
Sex	0.65 (0.52‐0.81)	<.001	0.80 (0.66‐0.98)	.03	1.24 (0.94‐1.63)	.13
CBCL internalizing (baseline)	0.51 (0.44‐0.58)	<.001	0.80 (0.72‐0.89)	<.001	1.28 (1.13‐1.44)	<.001
ICU^c^ total (baseline)	0.6 (0.5‐0.7)	<.001	0.75 (0.67‐0.83)	<.001	1.24 (1.09‐1.41)	.001
Duration of difficulties						
6‐12 months vs <6 months	0.75 (0.57‐0.99)	.045	0.79 (0.61‐1.03)	.08	0.96 (0.65‐1.41)	.84
>12 months vs <6 months	0.45 (0.34‐0.58)	<.001	0.66 (0.53‐0.84)	<.001	1.14 (0.81‐1.61)	.44
Severity at screening						
Definite versus minor	0.45 (0.36‐0.56)	<.001	0.66 (0.54‐0.81)	<.001	2.35 (1.67‐3.29)	<.001
Severe versus minor	0.43 (0.27‐0.68)	<.001	0.57 (0.40‐0.83)	.003	4.40 (2.87‐6.75)	<.001
Parent or family characteristics						
Family structure						
One biological parent versus 2 biological parents	0.81 (0.52‐1.26)	.36	0.80 (0.54‐1.19)	.27	1.16 (0.74‐1.84)	.52
Nonbiological parents versus 2 biological parents	0.91 (0.49‐1.70)	.78	0.92 (0.52‐1.63)	.78	1.06 (0.52‐2.15)	.88
Mother’s age group (years)						
≤26 versus 26‐40	1.09 (0.73‐1.62)	.67	0.91 (0.63‐1.30)	.59	1.02 (0.65‐1.61)	.94
>40 versus 26‐40	1.01 (0.49‐2.08)	.99	0.40 (0.18‐0.87)	.02	0.79 (0.30‐2.11)	.64
Father’s age group (years)						
≤26 versus 26‐40	1.54 (0.94‐2.54)	.09	1.68 (1.07‐2.66)	.03	1.55 (0.89‐2.72)	.12
>40 versus 26‐40	0.86 (0.56‐1.32)	.49	1.21 (0.84‐1.75)	.31	0.90 (0.54‐1.50)	.68
Mother’s education level						
Basic education versus university	1.69 (0.54‐5.34)	.37	1.60 (0.53‐4.83)	.41	2.79 (0.87‐8.92)	.08
Secondary education versus university	0.90 (0.66‐1.24)	.53	1.02 (0.76‐1.37)	.88	0.95 (0.64‐1.40)	.78
Upper degree in applied sciences versus a university degree	1.04 (0.79‐1.36)	.80	1.13 (0.88‐1.45)	.36	0.95 (0.68‐1.34)	.79
Father’s education level						
Basic education versus university	1.07 (0.62‐1.86)	.81	0.89 (0.52‐1.51)	.66	1.02 (0.51‐2.03)	.95
Secondary education versus university	0.79 (0.58‐1.06)	.12	0.91 (0.69‐1.20)	.49	1.12 (0.77‐1.65)	.55
Upper degree in applied sciences versus a university degree	0.79 (0.58‐1.07)	.12	0.82 (0.62‐1.09)	.16	1.07 (0.72‐1.57)	.75
Psychometric scales						
Parenting Scale total score	0.80 (0.71‐0.90)	<.001	0.90 (0.81‐1.01)	.07	0.86 (0.75‐1.00)	.047
DASS^d^-depression	0.93 (0.79‐1.09)	.35	0.98 (0.86‐1.12)	.77	1.06 (0.90‐1.24)	.51
DASS-anxiety	1.11 (0.95‐1.30)	.20	1.03 (0.90‐1.18)	.63	1.21 (1.04‐1.41)	.01
DASS-stress	0.66 (0.56‐0.77)	<.001	0.88 (0.76‐1.02)	.09	1.17 (0.97‐1.40)	.10
Adverse life events						
1 versus none	0.85 (0.65‐1.11)	.24	0.84 (0.66‐1.07)	.15	1.01 (0.73‐1.38)	.98
At least 2 versus none	0.87 (0.52‐1.44)	.58	1.14 (0.74‐1.75)	.55	1.13 (0.68‐1.91)	.63

a CBCL: Child Behavior Checklist 1.5-5.

b OR: odds ratio.

c ICU: Inventory of Callous-Unemotional Traits.

d DASS: 21-Item Depression, Anxiety and Stress Scale.

The final multinomial analysis of the effects of child, parent, or family, and program characteristics on the CBCL externalizing change categories is presented in Table 3. The statistically significant predictors related to child characteristics that were associated with limited improvement were the child being a male, internalizing symptoms, higher callous-unemotional traits, longer duration of difficulties, and higher severity at screening. The significant parent or family variables associated with reduced improvement included mother’s age >40 years, anxiety, and stress. Higher scores in dysfunctional parenting practices (PS) were associated with a lower likelihood of large improvement. The year of program completion, 2021, was associated with a lesser likelihood of a large or moderate improvement.

**Table 3.** Final multinomial model of the associations of all the predictors on the standardized change in CBCL^a^ externalizing as categorized into large improvement, moderate improvement, no change (reference group, n=851), and deterioration at 6 months after a parent training intervention targeting disruptive behavior in those aged 4 years in real-world settings, 2015‐2022. Missing observations per variable: sex (n=18), duration (n=45), adverse life events (n=239), family structure (n=10), maternal age (n=26), paternal age (n=117), maternal education (n=23), and paternal education (n=160).

Variable	Large improvement (n=833), OR^b^ (95% CI)	*P* value	Moderate improvement (n=1103), OR (95% CI)	*P* value	Deterioration (n=417), OR (95% CI)	*P* value
Child characteristics						
Boy versus girl	0.65 (0.52‐0.82)	<.001	0.80 (0.6‐0.98)	.03	1.26 (0.95‐1.68)	.11
CBCL internalizing (baseline)	0.54 (0.47‐0.63)	<.001	0.80 (0.72‐0.90)	<.001	1.15 (1.01‐1.30)	.04
ICU^c^ total (baseline)	0.64 (0.57‐0.73)	<.001	0.77 (0.69‐0.86)	<.001	1.32 (1.15‐1.51)	<.001
Duration of difficulties (months)						
6‐12 versus <6	0.75 (0.56‐0.99)	.04	0.80 (0.62‐1.05)	.10	0.92 (0.62‐1.37)	.67
>12 versus <6	0.43 (0.33‐0.56)	<.001	0.65 (0.51‐0.83)	<.001	1.09 (0.76‐1.55)	.65
Severity at screening						
Definite versus minor	0.45 (0.35‐0.56)	<.001	0.68 (0.55‐0.83)	<.001	2.29 (1.62‐3.24)	<.001
Severe versus minor	0.48 (0.30‐0.78)	.003	0.62 (0.43‐0.91)	.01	4.38 (2.80‐6.85)	<.001
Parent or family characteristics						
Mother’s age group (years)						
≤26 versus 26‐40	1.30 (0.88‐1.94)	.19	1.00 (0.70‐1.43)	.98	0.90 (0.57‐1.43)	.66
>40 versus 26‐40	1.09 (0.51‐2.31)	.83	0.40 (0.19‐0.86)	.02	0.97 (0.40‐2.36)	.95
Father’s age group (years)						
≤26 versus 26‐40	1.11 (0.67‐1.82)	.69	1.46 (0.94‐2.27)	.09	1.67 (0.96‐2.91)	.07
>40 versus 26‐40	0.82 (0.53‐1.27)	.38	1.22 (0.85‐1.75)	.27	0.87 (0.53‐1.45)	.60
Mother’s education level						
Basic education versus university	1.14 (0.41‐3.20)	.80	0.94 (0.36‐2.48)	.91	2.31 (0.85‐6.23)	.10
Secondary education versus university	0.75 (0.56‐1.01)	.054	0.91 (0.70‐1.17)	.46	1.0 (0.71‐1.40)	.99
Upper degree in applied sciences versus a university degree	0.93 (0.72‐1.21)	.59	1.04 (0.82‐1.31)	.75	1.10 (0.77‐1.44)	.74
Parenting Scale total score	0.87 (0.77‐1.00)	.03	0.94 (0.85‐1.05)	.27	0.90 (0.78‐1.03)	.13
DASS^d^-anxiety	1.10 (0.94‐1.28)	.26	1.02 (0.90‐1.17)	.74	1.20 (1.04‐1.39)	.02
DASS-stress	0.78 (0.67‐0.91)	.002	0.96 (0.84‐1.09)	.50	1.10 (0.94‐1.30)	.24
Program characteristics						
Year of completion						
2018‐10 March 2020 versus 2015‐2017	0.75 (0.54‐1.06)	.10	0.83 (0.61‐1.12)	.21	1.20 (0.79‐1.84)	.39
11 MAR–31 DEC 2020 versus 2015‐17	0.69 (0.46‐1.03)	.07	0.75 (0.53‐1.08)	.12	1.08 (0.65‐1.78)	.77
2021 versus 2015‐2017	0.66 (0.45‐0.97)	.03	0.69 (0.49‐0.97)	.03	1.13 (0.70‐1.83)	.61
2022‐2023 versus 2015‐2017	1.00 (0.69‐1.44)	.99	0.87 (0.62‐1.20)	.39	1.17 (0.74‐1.85)	.51
Not completed versus 2015‐2017	1.18 (0.63‐2.20)	.60	1.07 (0.61‐1.87)	.82	0.97 (0.43‐2.21)	.95

a CBCL: Child Behavior Checklist 1.5-5.

b OR: odds ratio.

c ICU: Inventory of Callous-Unemotional Traits.

d DASS: 21-Item Depression, Anxiety and Stress Scale.

## Discussion

### Principal Findings

This study, including 3204 families screened out of 49,504 families participating in annual medical check-ups for children aged 4 years, represents the largest implementation study to date that has examined the factors predicting the outcome of a parent training program. This study is unique in its use of population-based screening and its exceptionally large implementation sample. The findings apply to real-world contexts, and they have significant implications for public health when planning early interventions for children with disruptive behavior problems. First, 77% (2468/3204) of the children showed a successful program outcome at 6-month follow-up. Second, the severity and duration of the children’s problems at baseline were the strongest predictors for limited improvement. Third, parental anxiety or stress at baseline predicted limited improvement. Finally, program completion during the COVID-19 pandemic was associated with a reduced likelihood of improvement.

Overall, a clear majority of children in this study showed successful program outcomes at 6 months, which is in line with earlier implementation studies of the FSFP [27]. Literature on parent training remains limited in studies conducted in an implementation context where data are analyzed based on the type of outcome [5-7]. Such research is essential for a comprehensive understanding of the mechanisms and factors underlying the changes induced by a parent training program. For example, it is possible that the overall efficacy of parent training interventions is driven by a subgroup of children who show significant improvement [41]. However, in our study, 13% of the children showed a clear deterioration at 6-month follow-up. This group may reflect intervention resistance, indicating a need for different or additional support measures. Another possibility is that the 6-month follow-up after baseline—about 3 months postintervention—was too short to capture longer-term benefits. Future research should expand beyond group-level results by assessing patterns behind different types of outcomes.

### Child-Related Factors

As expected, there were a number of factors that influenced the levels of outcome, but overall, child characteristics had the strongest effect. Boys had a lower likelihood of large improvement and a higher likelihood of deterioration compared to girls, which suggests that girls responded to the program better than boys. Higher internalizing symptoms and callous-unemotional traits at baseline were associated with a lower likelihood of a favorable treatment response. Previously, callous-unemotional traits have been linked to poor treatment outcomes in family interventions for conduct problems [42]. Children with severe and definite initial behavioral difficulties had a fourfold and twofold risk of deterioration compared to those with minor difficulties, respectively. A longer duration of initial difficulties was associated with a lower likelihood of a favorable outcome, but not with a higher likelihood of a poor outcome. It is also plausible that experiencing difficulties over a prolonged period contributes to establishing unhelpful functioning and interaction patterns within the family system, which may require additional resources to change [43]. These findings highlight the importance of early identification of child disruptive behavior and tailored interventions designed to address each child’s specific needs. A marked presence of psychological maladjustment, along with a longer duration and greater severity of behavioral problems, can reflect psychopathological processes that may require different or additional forms of interventions.

### Parent and Family Factors

Parental stress and anxiety symptoms were also associated with outcomes. This may happen through a variety of mechanisms. For example, stress is linked to impaired, altered, and rigid memory functions [44], which may hinder parents’ ability to learn new skills. Greater parental mentalization skills are linked with lower externalizing behavior in their children [45]; however, anxiety is associated with deficits in mentalization [46]. Effective parenting requires the regulation of both internal (the parents’) and external (the child’s) emotional states [47,48], but psychopathological processes, again, are typically characterized by deficits in emotion regulation [49]. It may be beneficial to remain attentive to the psychosocial well-being of the parents throughout the course of a parent training program. Additionally, incorporating targeted supportive measures for parents with psychopathological symptoms at the starting phase of a parent training program may enhance treatment outcomes [50]. Such measures could reduce symptomatology, thereby making the training seem less demanding and more manageable for the parents. This, in turn, could facilitate more efficient skill acquisition and make the training more rewarding, thereby improving training outcomes and reducing dropouts [50].

The results indicated that maternal age over 40 years was linked to a decreased likelihood of moderate improvement. All other associations between parental age and outcomes were no longer significant in the final multinomial model. Although factors such as family structure and parents’ education level were individually predictive, they lost significance when tested alongside other factors. This reflects earlier findings [41] and suggests that the intervention is adaptable to diverse family backgrounds, irrespective of socioeconomic factors, which is an important element of equity in service quality and accessibility.

It should be noted that our findings in this study and previous literature show some key differences. The results derived from individual participant data meta-analyses [51-55] showed that the effects of the Incredible Years parenting intervention on disruptive behavior did not differ among families with different social and socioeconomic disadvantage, families with different styles of parenting, and children with different comorbidities, and severity of problems, sibling conflict, and parental depression were linked to stronger intervention effects. Several factors may explain these differences: the Incredible Years intervention was delivered in group format and face-to-face, and the samples spanned larger age ranges (from 1‐2 y up to 10‐12 y) [51-55]. In the present study, we used a standardized measure of individual change to mitigate possible floor effects and to show the individual change relative to baseline to identify factors predicting poor and favorable program outcomes.

### Program-Related Factors

Lastly, regarding program characteristics, completion during 2021 was linked to decreased improvement compared to completion during 2015‐2017. This may reflect additional stressors that families were experiencing during the COVID-19 public health restrictions (eg, lockdowns). Although these public health measures were introduced early in the pandemic in 2020, they persisted throughout 2021 [56]. Such stressors may have caused cumulative strain on parents and their children, potentially affecting parents’ ability to engage in positive parenting practices as the restrictions were prolonged [57,58]. It could be, for example, that at the beginning of the pandemic, the novel situation may have been perceived as novel and engaging, and families generally had sufficient resources to adapt to the new reality. Over time, however, as the restrictions persisted, everyday life may have been perceived as increasingly stressful [59]. This could have compromised parents’ resources to support their children and to consistently and effectively implement the FSFP skills training during the later stages of the pandemic compared to earlier phases.

The implementation of the FSFP parent training program involves integrating an empirically supported intervention into the health care service system. The FSFP has been implemented for over a decade [27] and is aligned with the existing service structure, which supports its feasibility and sustainability. Of note, the program completion rate was relatively high. We believe the most important contributing factors are the proactive screening process that identifies and engages parents at the onset of difficulties, and the fact that the intervention is not an unguided digital intervention. The intervention includes weekly human support from a designated family coach with whom the parent can build a working alliance. The family coach helps motivate and encourage the parent, clarify the content, support with challenging situations, and support the skills training. This likely translates into visible results in the family’s everyday life, which further encourages the participation. The remote delivery format is again likely to remove certain access barriers.

### Limitations

Several limitations of this study warrant discussion. The grouping of participants according to the type of outcome was based on statistical differences. While the statistics-based metric of change is commonly used and based on psychological theory, it may neither accurately capture the practical or clinical significance of these differences [60], nor reflect the participants’ subjective perceptions of the outcomes. Further, the outcome and child psychopathology measures are based solely on parent reports. It is possible that parental psychopathology affected their perceptions and appraisals of the child’s behavior [61]. Conversely, for young children who are under school age, the availability of other informants, such as teachers, is limited, and self-reports by children may not be a feasible option. Another limitation involves the participants for whom 6-month follow-up data were unavailable. We considered conducting multiple imputation; however, a comparative analysis between the group that provided 6-month data and the group that did not (Multimedia Appendix 2) showed that the groups differed on a number of factors. The data were hence not missing at random, whereby imputation can introduce bias rather than correct for it [62]. As the number of participants who did not provide data at the 6-month follow-up was relatively low (under 20%), we believe it would not cause a profound impact on the results. Participants who did not provide 6-month data tended to have completed fewer than 7 themes of the program, be single-parent families, have a lower parental age and education level, have experienced more adverse events, and exhibit higher scores on the depression, stress, and anxiety scales. However, given the large sample size, even small differences may have reached statistical significance.

The lack of a control group prevents us from drawing definite conclusions about the real-world effectiveness and the outcomes of the implementation strategy. However, we have previously shown in an RCT that the FSFP intervention was effective up to 24 months of follow-up [63]. In our previous study conducted in an implementation setting (n=600), we found that the 6-month follow-up results did not differ from the RCT study [27]. At the same time, we acknowledge that spontaneous improvement takes place among children exhibiting disruptive behavior [64], and this could have been a factor in some of the improvements observed in the current study.

As for the generalizability of the sample, the screening for the program is based on population-based screening. The participating child health clinics are geographically distributed across the whole country. A previous report [65] examining enrollment in the FSFP program showed that enrollment was associated with more severe and longer-term levels of disruptive behavior and comorbid emotional problems. Lower parental education and younger paternal age were associated with nonenrollment.

### Conclusions

The questions of which treatment works for whom, for which difficulties, under which circumstances, and delivered by whom were first raised long ago [66]. This study’s contribution to the field includes answering this question by identifying characteristics that may influence intervention performance in real-world settings, thereby allowing for more tailored interventions. An implication of our results in the real world is that children with longer-term and more severe behavioral symptoms may require more individualized approaches, and additional support may be necessary for parents experiencing elevated stress and anxiety. Such efforts could help ensure that more families can receive the optimal benefit from evidence-based parenting interventions.

Our work also broadens the methodological landscape by examining standardized individual change, which is an approach that has been used sparingly in previous parent training implementation studies. Our study further differs from much existing literature on parent training, which, to a large degree, is based on RCTs, while the literature on the implementation of parenting programs in community settings is scarcer. More studies expanding the existing RCT literature by showing factors influencing treatment outcome in real-world settings are needed to help guide parents and children to the right interventions.

## Supplementary material

10.2196/79592 Multimedia Appendix 1 Univariate analyses of the associations of individual predictors on the standardized change in CBCL externalizing, as categorized into large improvement, moderate improvement, no change (reference group), and decline. CBCL: Child Behavior Checklist 1.5-5.

10.2196/79592 Multimedia Appendix 2 Comparisons of child and family characteristics between those completing both baseline and 6-month follow-up (n=3204) and those completing only baseline (n=707) questionnaire.

## Abbreviations

CBCL: Child Behavior Checklist 1.5-5
DASS-21: 21-Item Depression, Anxiety and Stress Scale
FSFP: Finnish Strongest Families Parent
ICU: Inventory of Callous-Unemotional Traits
OR: odds ratio
PS: Parenting Scale
RCT: randomized controlled trial
SDQ: Strengths and Difficulties Questionnaire

## References

[1] Dretzke J, Frew E, Davenport C (2005). The effectiveness and cost-effectiveness of parent training/education programmes for the treatment of conduct disorder, including oppositional defiant disorder, in children. Health Technol Assess.

[2] Weber L, Kamp-Becker I, Christiansen H, Mingebach T (2019). Treatment of child externalizing behavior problems: a comprehensive review and meta-meta-analysis on effects of parent-based interventions on parental characteristics. Eur Child Adolesc Psychiatry.

[3] Scott S, Dadds MR (2009). Practitioner review: when parent training doesn’t work: theory‐driven clinical strategies. Child Psychology Psychiatry.

[4] Tolin DF, McKay D, Forman EM, Klonsky ED, Thombs BD (2015). Empirically supported treatment: recommendations for a new model. Clin Psychol Sci Pract.

[5] Lansford JE, Betancourt TS, Boller K (2022). The future of parenting programs: II implementation. Parent Sci Pract.

[6] Romano M, Schnurr M (2022). Mind the gap: strategies to bridge the research-to-practice divide in early intervention caregiver coaching practices. Topics Early Child Spec Educ.

[7] Michelson D, Davenport C, Dretzke J, Barlow J, Day C (2013). Do evidence-based interventions work when tested in the “real world?” A systematic review and meta-analysis of parent management training for the treatment of child disruptive behavior. Clin Child Fam Psychol Rev.

[8] Nock MK, Kazdin AE, Hiripi E, Kessler RC (2007). Lifetime prevalence, correlates, and persistence of oppositional defiant disorder: results from the National Comorbidity Survey Replication. J Child Psychol Psychiatry.

[9] DeRubeis RJ (2019). The history, current status, and possible future of precision mental health. Behav Res Ther.

[10] Reyno SM, McGrath PJ (2006). Predictors of parent training efficacy for child externalizing behavior problems--a meta-analytic review. J Child Psychol Psychiatry.

[11] Gardner F, Connell A, Trentacosta CJ, Shaw DS, Dishion TJ, Wilson MN (2009). Moderators of outcome in a brief family-centered intervention for preventing early problem behavior. J Consult Clin Psychol.

[12] McGilloway S, Mhaille GN, Bywater T (2012). A parenting intervention for childhood behavioral problems: a randomized controlled trial in disadvantaged community-based settings. J Consult Clin Psychol.

[13] Stolk MN, Mesman J, van Zeijl J (2008). Early parenting intervention: family risk and first-time parenting related to intervention effectiveness. J Child Fam Stud.

[14] Van Zeijl J, Mesman J, Van IJzendoorn MH (2006). Attachment-based intervention for enhancing sensitive discipline in mothers of 1- to 3-year-old children at risk for externalizing behavior problems: a randomized controlled trial. J Consult Clin Psychol.

[15] Baker S, Sanders MR (2017). Predictors of program use and child and parent outcomes of a brief online parenting intervention. Child Psychiatry Hum Dev.

[16] Day JJ, Baker S, Dittman CK (2021). Predicting positive outcomes and successful completion in an online parenting program for parents of children with disruptive behavior: an integrated data analysis. Behav Res Ther.

[17] Engelbrektsson J, Salomonsson S, Högström J, Sorjonen K, Sundell K, Forster M (2023). Is internet-based parent training for everyone? Predictors and moderators of outcomes in group vs. internet-based parent training for children with disruptive behavior problems. Behav Res Ther.

[18] Pokorna N, Palmer M, Pearson O (2024). Moderators of the effects of a digital parenting intervention on child conduct and emotional problems implemented during the COVID-19 pandemic: results from a secondary analysis of data from the supporting parents and kids through lockdown experiences (SPARKLE) randomized controlled trial. JMIR Pediatr Parent.

[19] Shelleby EC, Shaw DS (2014). Outcomes of parenting interventions for child conduct problems: a review of differential effectiveness. Child Psychiatry Hum Dev.

[20] Baumel A, Pawar A, Kane JM, Correll CU (2016). Digital parent training for children with disruptive behaviors: systematic review and meta-analysis of randomized trials. J Child Adolesc Psychopharmacol.

[21] Fleming GE, Kimonis ER, Furr JM, Comer JS (2020). Internet-delivered parent training for preschoolers with conduct problems: do callous-unemotional traits moderate efficacy and engagement?. J Abnorm Child Psychol.

[22] Leijten P, Raaijmakers MAJ, de Castro BO, Matthys W (2013). Does socioeconomic status matter? A meta-analysis on parent training effectiveness for disruptive child behavior. J Clinical Child Adolesc Psychol.

[23] Beauchaine TP, Webster-Stratton C, Reid MJ (2005). Mediators, moderators, and predictors of 1-year outcomes among children treated for early-onset conduct problems: a latent growth curve analysis. J Consult Clin Psychol.

[24] Gardner F, Hutchings J, Bywater T, Whitaker C (2010). Who benefits and how does it work? Moderators and mediators of outcome in an effectiveness trial of a parenting intervention. J Clin Child Adolesc Psychol.

[25] Furlong M, McGilloway S (2015). The longer term experiences of parent training: a qualitative analysis. Child Care Health Dev.

[26] Sourander A, McGrath PJ, Ristkari T (2016). Internet-assisted parent training intervention for disruptive behavior in 4-year-old children: a randomized clinical trial. JAMA Psychiatry.

[27] Sourander A, Ristkari T, Kurki M (2022). Effectiveness of an internet-based and telephone-assisted training for parents of 4-year-old children with disruptive behavior: implementation research. J Med Internet Res.

[28] Goodman R (2001). Psychometric properties of the strengths and difficulties questionnaire. J Am Acad Child Adolesc Psychiatry.

[29] Ilola AM, Lempinen L, Huttunen J, Ristkari T, Sourander A (2016). Bullying and victimisation are common in four-year-old children and are associated with somatic symptoms and conduct and peer problems. Acta Paediatr.

[30] Guyatt GH, Osoba D, Wu AW, Wyrwich KW, Norman GR, Clinical Significance Consensus Meeting Group (2002). Methods to explain the clinical significance of health status measures. Mayo Clin Proc.

[31] Achenbach TM, Rescorla LA (2000). Manual for the ASEBA Preschool Forms and Profiles.

[32] Essau CA, Sasagawa S, Frick PJ (2006). Callous-unemotional traits in a community sample of adolescents. Assessment.

[33] Ezpeleta L, de la Osa N, Granero R, Penelo E, Domènech JM (2013). Inventory of callous-unemotional traits in a community sample of preschoolers. J Clin Child Adolesc Psychol.

[34] Arnold DS, O’Leary SG, Wolff LS, Acker MM (1993). The parenting scale: a measure of dysfunctional parenting in discipline situations. Psychol Assess.

[35] Lovibond PF, Lovibond SH (1995). The structure of negative emotional states: comparison of the Depression Anxiety Stress Scales (DASS) with the Beck Depression and Anxiety Inventories. Behav Res Ther.

[36] Sinclair SJ, Siefert CJ, Slavin-Mulford JM, Stein MB, Renna M, Blais MA (2012). Psychometric evaluation and normative data for the Depression, Anxiety, and Stress Scales-21 (DASS-21) in a nonclinical sample of U.S. adults. Eval Health Prof.

[37] Leijten P, Gardner F, Melendez-Torres GJ (2019). Meta-analyses: key parenting program components for disruptive child behavior. J Am Acad Child Adolesc Psychiatry.

[38] Norman GR, Sloan JA, Wyrwich KW (2003). Interpretation of changes in health-related quality of life: the remarkable universality of half a standard deviation. Med Care.

[39] Mouelhi Y, Jouve E, Castelli C, Gentile S (2020). How is the minimal clinically important difference established in health-related quality of life instruments? Review of anchors and methods. Health Qual Life Outcomes.

[40] Korda H (2013). Bringing evidence-based interventions to the field: the fidelity challenge. J Public Health Manag Pract.

[41] Leijten P, Raaijmakers M, Wijngaards L (2018). Understanding who benefits from parenting interventions for children’s conduct problems: an integrative data analysis. Prev Sci.

[42] Hawes DJ, Price MJ, Dadds MR (2014). Callous-unemotional traits and the treatment of conduct problems in childhood and adolescence: a comprehensive review. Clin Child Fam Psychol Rev.

[43] Carr A (2012). Family Therapy: Concepts, Process and Practice.

[44] Sandi C (2013). Stress and cognition. Wiley Interdiscip Rev Cogn Sci.

[45] Nieto-Retuerto M, Torres-Gomez B, Alonso-Arbiol I (2025). Parental mentalization and children’s externalizing problems: a systematic review and meta-analysis. Dev Psychopathol.

[46] Chevalier V, Simard V, Achim J (2023). Meta-analyses of the associations of mentalization and proxy variables with anxiety and internalizing problems. J Anxiety Disord.

[47] Casline E, Patel ZS, Timpano KR, Jensen-Doss A (2021). Exploring the link between transdiagnostic cognitive risk factors, anxiogenic parenting behaviors, and child anxiety. Child Psychiatry Hum Dev.

[48] Rutherford HJV, Wallace NS, Laurent HK, Mayes LC (2015). Emotion regulation in parenthood. Dev Rev.

[49] Aldao A, Nolen-Hoeksema S, Schweizer S (2010). Emotion-regulation strategies across psychopathology: a meta-analytic review. Clin Psychol Rev.

[50] Maliken AC, Katz LF (2013). Exploring the impact of parental psychopathology and emotion regulation on evidence-based parenting interventions: a transdiagnostic approach to improving treatment effectiveness. Clin Child Fam Psychol Rev.

[51] Gardner F, Leijten P, Mann J (2017). Public Health Res.

[52] Gardner F, Leijten P, Melendez-Torres GJ (2019). The earlier the better? Individual participant data and traditional meta-analysis of age effects of parenting interventions. Child Dev.

[53] Gardner F, Leijten P, Harris V (2019). Equity effects of parenting interventions for child conduct problems: a pan-European individual participant data meta-analysis. Lancet Psychiatry.

[54] Leijten P, Scott S, Landau S (2020). Individual participant data meta-analysis: impact of conduct problem severity, comorbid attention-deficit/hyperactivity disorder and emotional problems, and maternal depression on parenting program effects. J Am Acad Child Adolesc Psychiatry.

[55] Melendez-Torres GJ, Leijten P, Scott S (2024). Disruptive child behavior severity and parenting program session attendance: Individual participant data meta-analysis. J Consult Clin Psychol.

[56] (2021). Government resolution STM/2021/170: government resolution on an action plan implementing the hybrid strategy to manage the COVID-19 epidemic. Finnish Government.

[57] Chung G, Tilley JL, Netto N, Chan A, Lanier P (2024). Parenting stress and its impact on parental and child functioning during the COVID-19 pandemic: a meta-analytical review. Int J Stress Manag.

[58] Connell CM, Strambler MJ (2021). Experiences with COVID-19 stressors and parents’ use of neglectful, harsh, and positive parenting practices in the Northeastern United States. Child Maltreat.

[59] Friedmann A, Buechel C, Seifert C, Eber S, Mall V, Nehring I (2023). Easing pandemic-related restrictions, easing psychosocial stress factors in families with infants and toddlers? Cross-sectional results of the three wave CoronabaBY study from Germany. Child Adolesc Psychiatry Ment Health.

[60] Jacobson NS, Roberts LJ, Berns SB, McGlinchey JB (1999). Methods for defining and determining the clinical significance of treatment effects: description, application, and alternatives. J Consult Clin Psychol.

[61] Callender KA, Olson SL, Choe DE, Sameroff AJ (2012). The effects of parental depressive symptoms, appraisals, and physical punishment on later child externalizing behavior. J Abnorm Child Psychol.

[62] Curnow E, Tilling K, Heron JE, Cornish RP, Carpenter JR (2023). Multiple imputation of missing data under missing at random: including a collider as an auxiliary variable in the imputation model can induce bias. Front Epidemiol.

[63] Sourander A, McGrath PJ, Ristkari T (2018). Two-year follow-up of internet and telephone assisted parent training for disruptive behavior at age 4. J Am Acad Child Adolesc Psychiatry.

[64] López-Romero L, Romero E, Andershed H (2015). Conduct problems in childhood and adolescence: developmental trajectories, predictors and outcomes in a six-year follow up. Child Psychiatry Hum Dev.

[65] Lintula S, Sourander A, Hinkka-Yli-Salomäki S (2025). Enrollment and completion rates of a nationwide guided digital parenting program for children with disruptive behavior before and during COVID-19. Eur Child Adolesc Psychiatry.

[66] Hofmann SG (2025). A network control theory of dynamic systems approach to personalize therapy. Behav Ther.

