# Supplementary material for: Predictors of Outcomes of Parent Training Targeting Disruptive Behavior in Children Aged 4 Years at 6-Month Follow-Up: Results From a Large Prospective Cohort Implementation Study
Source: J Med Internet Res. 2026 Apr 28;28:e79592. doi: 10.2196/79592 (PMC13123636; doi:10.2196/79592)
Supplement: Multimedia Appendix 1 — Univariate analyses of the associations of individual predictors on the standardized change in CBCL externalizing, as categorized into large improvement, moderate improvement, no change (reference group), and decline. CBCL: Child Behavior Checklist 1.5-5. [file jmir-v28-e79592-s001.docx]

|  |  | Large improvement  (n = 828) | | | |  | Moderate improvement (n = 1099) | | | |  | Decline  (n = 413) | | | |
| --- | --- | --- | --- | --- | --- | --- | --- | --- | --- | --- | --- | --- | --- | --- | --- |
|  |  |  |  |  |  |  |  |  |  |  |  |  |  |  |  |
|  |  | OR | 95% CI | | *p* |  | OR | 95% CI | | *p* |  | OR | 95% CI | | *p* |
| ***Child characteristics*** |  |  |  |  |  |  |  |  |  |  |  |  |  |  |  |
| Male vs. Female |  | 0.63 | 0.52 | 0.77 | <.001 |  | 0.76 | 0.63 | 0.91 | .004 |  | 1.28 | 0.99 | 1.66 | .062 |
| CBCL Internalizing (baseline) |  | 0.39 | 0.34 | 0.44 | <.001 |  | 0.70 | 0.63 | 0.77 | <.001 |  | 1.48 | 1.33 | 1.64 | <.001 |
| ICU Total (baseline) |  | 0.47 | 0.42 | 0.53 | <.001 |  | 0.67 | 0.61 | 0.74 | <.001 |  | 1.45 | 1.29 | 1.64 | <.001 |
| Duration of difficulties |  |  |  |  |  |  |  |  |  |  |  |  |  |  |  |
| 6-12 months vs. < 6 months |  | 0.54 | 0.41 | 0.69 | <.001 |  | 0.67 | 0.52 | 0.86 | .002 |  | 1.20 | 0.83 | 1.74 | .34 |
| > 12 months vs. < 6 months |  | 0.28 | 0.22 | 0.36 | <.001 |  | 0.53 | 0.43 | 0.66 | <.001 |  | 1.66 | 1.20 | 2.31 | .002 |
| Severity at screening |  |  |  |  |  |  |  |  |  |  |  |  |  |  |  |
| definite vs. minor |  | 0.32 | 0.26 | 0.39 | <.001 |  | 0.56 | 0.47 | 0.68 | <.001 |  | 2.70 | 1.95 | 3.74 | <.001 |
| severe vs. minor |  | 0.19 | 0.13 | 0.30 | <.001 |  | 0.40 | 0.28 | 0.56 | <.001 |  | 6.32 | 4.24 | 9.42 | <.001 |
| ***Parent/family characteristics*** |  |  |  |  |  |  |  |  |  |  |  |  |  |  |  |
| Family structure |  |  |  |  |  |  |  |  |  |  |  |  |  |  |  |
| one biological parent vs. two biological parents |  | 0.71 | 0.52 | 0.98 | .034 |  | 0.84 | 0.63 | 1.11 | .22 |  | 1.55 | 1.11 | 2.15 | .009 |
| non-biological parents vs. two biological parents |  | 0.91 | 0.54 | 1.55 | .74 |  | 0.88 | 0.53 | 1.44 | .60 |  | 1.31 | 0.72 | 2.39 | 0.37 |
| Mother's age group |  |  |  |  |  |  |  |  |  |  |  |  |  |  |  |
| ≤ 26 vs. 26-40 years |  | 1.09 | 0.82 | 1.45 | .54 |  | 1.09 | 0.83 | 1.42 | .54 |  | 1.53 | 1.11 | 2.12 | .010 |
| > 40 vs. 26-40 years |  | 0.83 | 0.46 | 1.49 | .53 |  | 0.56 | 0.31 | 1.02 | .06 |  | 0.92 | 0.45 | 1.88 | .81 |
| Father's age group |  |  |  |  |  |  |  |  |  |  |  |  |  |  |  |
| ≤ 26 vs. 26-40 years |  | 1.26 | 0.86 | 1.84 | .24 |  | 1.45 | 1.02 | 2.06 | .039 |  | 2.11 | 1.40 | 3.17 | <.001 |
| > 40 vs. 26-40 years |  | 0.86 | 0.60 | 1.24 | .41 |  | 1.08 | 0.78 | 1.50 | .64 |  | 0.98 | 0.63 | 1.53 | .94 |
| Mother's education level |  |  |  |  |  |  |  |  |  |  |  |  |  |  |  |
| basic education vs. university |  | 1.18 | 0.50 | 2.78 | .70 |  | 1.20 | 0.53 | 2.72 | .66 |  | 3.04 | 1.30 | 7.11 | .010 |
| secondary education vs. university |  | 0.85 | 0.67 | 1.08 | .17 |  | 1.02 | 0.81 | 1.27 | .89 |  | 1.28 | 0.96 | 1.71 | .09 |
| upper degree in applied sciences vs. university |  | 1.05 | 0.83 | 1.32 | .71 |  | 1.11 | 0.89 | 1.38 | .36 |  | 1.10 | 0.89 | 1.38 | .54 |
| Father's education level |  |  |  |  |  |  |  |  |  |  |  |  |  |  |  |
| basic education vs. university |  | 1.00 | 0.62 | 1.63 | 1.00 |  | 0.92 | 0.57 | 1.48 | .73 |  | 1.46 | 0.81 | 2.65 | .21 |
| secondary education vs. university |  | 0.79 | 0.62 | 1.00 | .05 |  | 0.95 | 0.75 | 1.19 | .63 |  | 1.35 | 0.99 | 1.85 | .06 |
| upper degree in applied sciences vs. university |  | 0.89 | 0.68 | 1.16 | .39 |  | 0.89 | 0.68 | 1.15 | .36 |  | 1.18 | 0.83 | 1.68 | .36 |
| Parenting scale total score |  | 0.69 | 0.63 | 0.76 | <.001 |  | 0.86 | 0.78 | 0.94 | .0009 |  | 0.99 | 0.88 | 1.12 | 0.88 |
| DASS-Depression |  | 0.67 | 0.60 | 0.75 | <.001 |  | 0.87 | 0.79 | 0.95 | .003 |  | 1.25 | 1.13 | 1.39 | <.001 |
| DASS-Anxiety |  | 0.75 | 0.66 | 0.84 | <.001 |  | 0.90 | 0.81 | 0.99 | .0288 |  | 1.31 | 1.19 | 1.46 | <.001 |
| DASS-Stress |  | 0.62 | 0.56 | 0.68 | <.001 |  | 0.86 | 0.79 | 0.95 | .002 |  | 1.34 | 1.20 | 1.50 | <.001 |
| Adverse life events |  |  |  |  |  |  |  |  |  |  |  |  |  |  |  |
| 1 vs. none |  | 0.79 | 0.62 | 1.00 | .046 |  | 0.81 | 0.65 | 1.01 | .06 |  | 1.19 | 0.90 | 1.57 | .22 |
| At least 2 vs. none |  | 0.53 | 0.35 | 0.82 | .004 |  | 0.90 | 0.64 | 1.28 | .57 |  | 1.49 | 0.98 | 2.27 | .06 |
| ***Program characteristics*** |  |  |  |  |  |  |  |  |  |  |  |  |  |  |  |
| Program completion |  |  |  |  |  |  |  |  |  |  |  |  |  |  |  |
| < 7 themes completed vs. at least 7 themes completed |  | 1.22 | 0.62 | 2.39 | .56 |  | 1.41 | 0.76 | 2.61 | .28 |  | 0.76 | 0.30 | 1.96 | .58 |
| Year of completion |  |  |  |  |  |  |  |  |  |  |  |  |  |  |  |
| 2018-10MAR 2020 vs. 2015-2017 |  | 0.78 | 0.58 | 1.06 | .11 |  | 0.80 | 0.60 | 1.06 | .12 |  | 1.24 | 0.84 | 1.83 | .29 |
| 11MAR-31DEC 2020 vs. 2015-2017 |  | 0.69 | 0.48 | 0.99 | .044 |  | 0.71 | 0.51 | 0.99 | .045 |  | 0.95 | 0.60 | 1.51 | .83 |
| 2021 vs. 2015-2017 |  | 0.72 | 0.51 | 1.01 | .057 |  | 0.70 | 0.51 | 0.96 | .026 |  | 1.10 | 0.71 | 1.69 | .68 |
| 2022-2023 vs. 2015-2017 |  | 0.93 | 0.68 | 1.29 | .68 |  | 0.82 | 0.60 | 1.11 | .19 |  | 1.15 | 0.75 | 1.75 | .53 |
| Not completed vs. 2015-2017 |  | 1.13 | 0.65 | 1.97 | .68 |  | 1.11 | 0.65 | 1.87 | .70 |  | 1.13 | 0.65 | 1.97 | .65 |
| Note: Reference group No change (n= 846). | | | |  |  |  |  |  |  |  |  |  |  |  |  |
