# Supplementary material for: Predictors of Outcomes of Parent Training Targeting Disruptive Behavior in Children Aged 4 Years at 6-Month Follow-Up: Results From a Large Prospective Cohort Implementation Study
Source: J Med Internet Res. 2026 Apr 28;28:e79592. doi: 10.2196/79592 (PMC13123636; doi:10.2196/79592)
Supplement: Multimedia Appendix 2 — Comparisons of child and family characteristics between those completing both baseline and 6-month follow-up (n=3204) and those completing only baseline (n=707) questionnaire. [file jmir-v28-e79592-s002.docx]

| **Variable** | **Completing baseline and**  **6-month follow-up**  **(n=3204)** | **Completing only baseline**  **(n=707)** | ***p*** |
| --- | --- | --- | --- |
| **Child characteristics** |  |  |  |
| Sex, n (%) |  |  | 0.29 |
| Female | 1158 (36.35) | 272 (38.47) |  |
| Male | 2028 (63.65) | 435 (61.53) |  |
| Difficulties, n (%) |  |  | 0.43 |
| Minor | 1407 (43.91) | 295 (41.73) |  |
| Definite | 1497 (46.72) | 337 (47.67) |  |
| Severe | 300 (9.36) | 75 (10.61) |  |
| Duration of difficulties, n (%) |  |  | 0.39 |
| 6 months | 934 (29.57) | 218 (31.55) |  |
| 6–12 months | 853 (27.00) | 192 (27.79) |  |
| >12 months | 1372 (43.43) | 281 (40.67) |  |
| Adverse life events, n (%) |  |  | <.001 |
| None | 2013 (67.89) | 471 (57.23) |  |
| 1 | 732 (24.69) | 251 (30.50) |  |
| ≥2 | 220 (7.42) | 101 (12.27) |  |
| Psychometric scales*,* mean (SD) |  |  |  |
| CBCL internalizing | -0.02 (0.99) | 0.07 (1.06) | 0.058 |
| ICU score | -0.003 (0.99) | 0.01 (1.05) | 0.73 |
| **Family characteristics** |  |  |  |
| Family structure, n (%) |  |  | <.001 |
| Two biological parents | 2721 (85.19) | 538 (76.53) |  |
| One biological parent | 362 (11.33) | 141 (20.06) |  |
| Two non-biological parents | 111 (3.48) | 24 (3.41) |  |
| Maternal age, n (%) |  |  | <.001 |
| <26 years | 444 (13.97) | 162 (23.21) |  |
| 26–40 years | 2657 (83.61) | 522 (74.79) |  |
| >40 years | 77 (2.42) | 14 (2.01) |  |
| Paternal age, n (%) |  |  | <.001 |
| <26 years | 261 (8.45) | 88 (13.50) |  |
| 26–40 years | 2574 (83.38) | 508 (77.91) |  |
| >40 years | 252 (8.16) | 56 (8.59) |  |
| Maternal education, n (%) |  |  | <.001 |
| Basic education | 50 (1.57) | 40 (5.71) |  |
| Secondary education | 951 (29.90) | 294 (41.94) |  |
| Upper degree in applied sciences | 1059 (33.29) | 194 (27.67) |  |
| University | 1121 (35.24) | 173 (24.68) |  |
| Paternal education, n (%) |  |  | <.001 |
| Basic education | 144 (4.73) | 48 (7.57) |  |
| Secondary education | 1346 (44.22) | 343 (54.10) |  |
| Upper degree in applied sciences | 757 (24.87) | 123 (19.40) |  |
| University | 797 (26.18) | 120 (18.93) |  |
| Psychometric scales, mean (SD) |  |  |  |
| Parenting scale | -0.02 (0.98) | 0.08 (1.07) | 0.037 |
| DASS-depression | -0.05 (0.97) | 0.21 (1.12) | <.001 |
| DASS-anxiety | -0.03 (0.95) | 0.14 (1.20) | <.001 |
| DASS-stress | -0.03 (0.98) | 0.14 (1.06) | <.001 |
| Themes completed |  |  |  |
| <7 themes | 70 (2.18) | 364 (51.49) | <.001 |
| ≥7 themes | 3134 (97.82) | 343 (48.51) |  |

Note. Missing values: sex = 220, severity of difficulties = 202, duration = 263, family structure = 216, paternal age = 374, maternal age = 237, paternal education = 435, maternal education = 231, themes = 202, adverse events = 325.
